# Comparing the Technological and Intraoperative Performances of Da Vinci xi and DaVinci 5 Robotic Platforms in Patients Undergoing Robotic-Assisted Radical Prostatectomy

**DOI:** 10.1590/S1677-5538.IBJU.2024.0569

**Published:** 2025-01-10

**Authors:** Ahmed Gamal, Marcio Covas Moschovas, Shady Saikali, Sumeet Reddy, Yu Ozawa, Rohan Sharma, Avaneesh Kunta, Travis Rogers, Vipul Patel

**Affiliations:** 1 AdventHealth Global Robotics Institute USA AdventHealth Global Robotics Institute, Celebration, USA; 2 University of Central Florida Orlando USA University of Central Florida (UCF), Orlando, USA

## Abstract

**Introduction:**

In the last two decades, several Da Vinci robotic platforms have been released in the market, revolutionizing the field of robotic-assisted surgery (1, 2). The system has seen numerous modifications, with several Da Vinci® robotic models being introduced, each featuring ongoing technological advancements in ergonomics, instrumentation, high-definition imaging, EndoWrist™ technology, and single-port surgery capabilities (3, 4). Building on this, the new generation Da Vinci 5 robot promises significant hardware and software improvements, with the potential for enhanced operative performance (2, 5). In this video, we will illustrate several technical advancements of the Da Vinci 5.

**Material and methods:**

*W*e performed a video compilation comparing the Da Vinci 5 and Da Vinci Xi during radical prostatectomy. The video will highlight the technical modifications of the new platform, showcasing the advancements and improvements in the Da Vinci 5 system. Additionally, this video will illustrate key aspects of the surgery, including anterior bladder neck access, lateral bladder dissection from the prostate, posterior prostate dissection and anastomosis.

**Surgical technique:**

We performed our RARP technique with our standard approach in all patients (6–8). With this new platform, we maintained our conventional technique without any modifications or adaptions from the trocar placement until anastomosis. The beginning of the case is performed as usual, we first identify the anterior bladder neck and then complete its dissection with Maryland and Scissors. Then, we proceed to the posterior bladder neck dissection, seminal vesicles control and nerve-sparing. In sequence, we control the prostate arterial pedicles with hem-o-lok clips and then we perform the apical dissection until dividing the urethra. Finally, we perform the hemostasis, posterior reconstruction (Rocco's technique) and anastomosis with barbed suture.

**Results:**

The Da Vinci 5 features several key upgrades. The first part of our video described the console, patient cart, and energy tower modifications. The console has been ergonomically redesigned for a flat neck posture to decrease muscle fatigue, and the handgrip now includes a rubber surface for better grip (9). The patient cart, similar to the previous generation, has updated helm interfaces and integrated commands with the console and vision tower. In sequence, we described the instrument modifications and the step-by-step technique showing the DV5 and DV-Xi. Force feedback instruments provide three degrees of tactile feedback, enhancing tissue manipulation. A new security system ensures instruments can only be inserted when clear of tissues and obstructions, reducing the risk of errors. Another modification regards the ability to switch instruments and camera.

**Conclusion:**

While using and evaluating the DV5 in more than 100 cases, we noticed some improvements in the ergonomics and digital interface. The intraoperative performance was similar among the platforms and all procedures were performed without intraoperative complications or problems with the system. However, we are still evaluating the long-term outcomes and potential clinical advantages provided by this new platform.

Available at: http://www.intbrazjurol.com.br/video-section/20240569_Gamal_et_al
